# Significance of Selected Posturographic Methods in Diagnosis of Balance Disorders in Patients with Early-Stage Gonarthrosis

**DOI:** 10.3390/jcm13113298

**Published:** 2024-06-03

**Authors:** Amanda Maria Kostro, Artur Augustynik, Anna Kuryliszyn-Moskal, Jacek Jamiołkowski, Monika Pocienè, Zofia Dzięcioł-Anikiej

**Affiliations:** 1 Rehabilitation Clinic, Medical University of Białystok, 15-089 Białystok, Polandanna.kuryliszyn-moskal@umb.edu.pl (A.K.-M.); 2 Population Medicine and Civilisation Disease Prevention Division, Medical University of Białystok, 15-089 Białystok, Poland; 3 Department of Physiotherapy and Beauty Therapy, Klaipèdos Valstybine Kolegia, 91274 Klaipėda, Lithuania

**Keywords:** gonarthrosis, posturography, functionality, diagnosis, physiotherapy

## Abstract

Degenerative joint disease is a dynamic pathological process characterised by a destabilisation of the degradation and synthesis processes of articular cartilage and subchondral bone layer. Studies suggest that individuals with gonarthrosis experience deficits in proprioception, in addition to changes within their joints, which directly affects their ability to maintain posture and increases their risk of falling. **Objectives:** The aim of this study was to assess the functional status of patients with gonarthrosis through a posturographic examination conducted on a stabilometric platform (force plate) and a functional clinical examination. **Methods:** Participants were divided into two groups—a control group (*n* = 125) and a study group (*n* = 125). During the qualification process, subjective and objective examinations were conducted, including a functional assessment by means of such tests as the “Up and Go” Test, Functional Reach Test, Five Time Sit to Stand Test, and the Step Test. Subsequently, an assessment was conducted on the force plate by means of a posturographic test—the Romberg test performed with open and closed eyes in a standing position—and balance was evaluated using the Berg Balance Scale. The obtained data were analysed with the use of the IBM SPSS Statistics software version 27.0, by means of the Mann–Whitney test, and correlations were determined by means of Spearman’s test. A significance level of *p* = 0.05 was adopted. **Results:** Statistically significant differences were observed among the assessed groups as a result of both functional and posturographic examinations, along with positive correlations for disease duration, age, and BMI index. **Conclusions:** Patients with gonarthrosis exhibited disturbances in balance, functionality, and posture compared to healthy individuals in the control group.

## 1. Introduction

Balance is associated with the motor ability of humans, which allows for the controlled maintenance of our centre of gravity within the support base plane [1]. Its main tasks include the control of mechanisms responsible for maintaining an upright posture and the position of the respective elements of the musculoskeletal system in space during various motor activities. That process requires collaboration with coordinative abilities such as movement differentiation, reaction speed, and spatial orientation [2]. The ability to maintain balance is ensured, among other factors, by a properly functioning nervous and muscular system through the reflexive tension of anti-gravity muscles known as postural muscles. Those muscles include the suboccipital, erector spinae, iliocostalis lumborum, gluteus maximus, triceps surae, tibialis anterior, quadriceps femoris, obliques, rectus abdominis, and sternocleidomastoid muscles [3]. 

Stability is a much broader concept than balance, and it is defined as the ability to actively restore an original body position which has been disrupted from its previously established state of equilibrium due to destabilising factors. It represents the human body’s response to emerging disturbances, including one’s own motor activity or external forces resulting from environmental variability or interactions with our surroundings [1,4]. Maintaining stability is associated with the proper activity of the nervous system, allowing for the anticipation of a destabilising situation. It occurs through the initiation of appropriate strategies to control balance [5].

Throughout life, both balance and stability undergo constant changes. During human development, differences are observed in both the position of the centre of gravity and the modification of the support base plane. Due to our bipedal body position, our centre of gravity is elevated from a low position, and our support base plane changes from a large one, encompassing the entire body, to a relatively small one defined as the surfaces of the feet. The ontogenetic process allows for the formation of movement patterns aimed at maintaining balance, including static reactions—postural reflexes, static–kinetic reactions—straightening reflexes, and balance reflexes—postural ones [6].

The system responsible for controlling the processes of balance and stability undergoes degradation with age. One consequence of these changes is an increased risk of falls in individuals over 65 years of age. The first signs of aging in the postural control system are observed around the age of 50. At this time, there is a visible decrease in the muscle mass of about 1–2% per year, resulting in a 40% loss of its maximum value by the age of 70–80. There are also disturbances in the responses to stimuli, and adaptive mechanisms operate with a delay. Additionally, difficulties in detecting small changes in the angle of the ankle joint, not exceeding 1 degree, are observed. This means that the nervous system has a limited ability to recognise joint positions, which affects a person’s centre of gravity in a standing position. Furthermore, research indicates that advanced age alters proprioception in the lower limbs, causing reduced sensitivity, acuity, and integrity of the proprioceptive signals, as evidenced by the postural sway expressed by the increased movement of the centre of pressure on support surfaces [6,7]. It is hypothesised that the deficit in postural stability, which progresses with age, may be caused by impairments in detecting a balance loss, as well as clumsiness and slowed movement. In older individuals, a lack of proper motor coordination has been observed, leading to a significant clumsiness in backward leaning attempts and a noticeable tendency to limit their stability range when leaning forward or to the side [8]. The aging process also adversely affects the musculoskeletal system, deteriorating posture and increasing the risk of falls and gait disturbances. The prevalence of degenerative processes over regenerative ones within the connective tissue disrupts homeostasis, which is essential for the functioning of many systems related not only to the musculoskeletal system but also to the nervous, vascular, and hormonal systems. Degenerative joint disease (DJD) plays a significant role in the aging process, causing the destruction of articular cartilage and exacerbating the formation of secondary changes in bone ends, which ultimately damage joints, impair their function, and are a source of pain. The knee joints are among those most frequently involved in the degenerative process (accounting for as much as 25% of occurrences). According to several sources, gonarthrosis, i.e., knee DJD, is one of the reasons why 10% of people over 55 years of age and about 30% of people over 65 years of age do not seek immediate treatment for injuries [9,10,11]. 

Gonarthrosis is a dynamic pathological process characterised by the destabilisation of the degradation and synthesis processes of articular cartilage and the subchondral bone layer. The disease affects all joint tissues, leading to softening, fibrous changes, ulcers, and a loss of articular cartilage, as well as sclerosis, a densification of subchondral bone tissue, and the formation of osteophytes and subchondral cysts. The clinical presentation of gonarthrosis primarily includes joint pain, a limited range of motion, and crepitus during movement, sometimes accompanied by synovial inflammation but without systemic symptoms [12]. Gonarthrosis is a progressive and chronic process, and treatment aims to slow down the progression of the disease, leading to the impaired mobility and instability of the affected joint, and ultimately disability, which can even lead to depression due to the patient’s inability to fulfil their previous social functions [13]. In the course of gonarthrosis, the most commonly affected joints are the knee joints, hip joints, and joints within the hands, as well as the cervical and lumbar spine [14]. The mechanism of these degenerative changes primarily results from the interaction of mechanical and biological factors. Mechanical factors include excessive loading due to the reduced articular cartilage surface area and unevenly distributed loads on the joint, which exert increased pressure on the articular surfaces during repetitive loading. In addition to the disruption of the joint’s biomechanics, physicochemical changes at the level of cartilage and joint tissues play an important role. The main cause of disease development is considered to be an imbalance between the processes of the synthesis and degradation of articular cartilage [15,16]. 

Studies suggest that individuals with gonarthrosis experience deficits in proprioception, in addition to changes within their joints, which directly affects their ability to maintain posture and increases their risk of falls. Moreover, balance and ability to stabilise the body during movement depend on a properly functioning somatosensory system and the correct coordination of proprioceptive, visual, and vestibular impulses [17]. In the case of gonarthrosis, muscle dysfunction is often observed, leading to reduced joint mobility and pain. Nociceptive impulses in the affected joints inhibit the activity of muscles and spinal nerves, directly affecting the condition of muscles supporting the joint and those located at a considerable distance from the painful area. This condition causes a kind of destabilisation of stabilising balance and worsens the flow of proprioceptive impulses. Research results suggest that disruption of these impulses leads to decreased tension in the periarticular muscles, resulting in an increased mechanical load on the joint. With age, there is a decrease in load-bearing capacity, and reduced physical activity due to pain leads to further muscle volume reduction and joint instability. Consequently, chronic pain leads to muscle atrophy, weakening, and an imbalance between antagonist and agonist muscle groups, affecting range of motion restrictions and worsening the condition of the periarticular tissues, contributing to the exacerbation of degenerative changes [10,18,19]. Therefore, it appears essential to implement methods for assessing balance in patients with osteoarthritis.

Among the tests used for assessing balance and motor coordination, the most common are clinical tests, including the Romberg test or the Unterberger test, and/or the Fukuda test. However, those methods are subjective, and their reliability depends on the skill of the examiner. On the other hand, among the numerous objective scales and tests available, the most frequently used are the Berg Balance Scale and Tinetti Test, the Up and Go Test, Functional Reach Test, One Leg Standing Test, Step Test, Fullerton Advance Balance (FAB) Scale, and Dynamic Gait Index (DGI). They involve motor tasks, and the result usually consists of the time taken by the patient to perform the task, the distance covered, or the sum of points for respective tasks. However, there are objective quantitative methods that allow for a sensitive and accurate analysis of the patient’s balance. Modern technological solutions are applied here, such as stabilometric platforms or mats (force plates) that provide a simple and quick, yet reliable, assessment of the patient’s stability while standing. A posturographic examination involves an involuntary displacement of the Centre of Pressure (COP) during a bipedal or single-leg stance with closed or open eyes [20,21,22,23,24,25,26,27,28,29,30,31].

### Aim

The aim of this study has been to assess the functional status of patients with gonarthrosis through a posturographic examination conducted on a stabilometric platform (force plate), as well as a functional clinical assessment. The following research hypothesis has been put forward: lower limb impairments are observed in patients with stage II knee osteoarthritis, as expressed in both clinical and posturographic examinations.

## 2. Methodology

This observational clinical study was conducted, after having obtained the approval number APK.002.103.2020 (approved on 25 February 2021) from the Bioethics Committee of the Medical University of Białystok, from February 2021 to December 2021. The sample size calculation was based on the estimated population of people with gonarthrosis in the city of Białystok, which was 289, with a fraction size of 0.5, a confidence interval of 95% and a statistical error of 5%. The number of 165 people was obtained. This study included patients who had appeared at the Rehabilitation Clinic of the Medical University of Białystok and the Emil Chojnowski Orthopaedic-Prosthetic Centre in Białystok. Before the commencement of the study, all participants had been informed about the purpose of the research and the methods used, and they had the opportunity to clarify all and any outstanding issues with the medical attendant, after which they provided their consent to participate by signing an appropriate statement. The inclusion criteria for the program included a medical diagnosis of bilateral knee osteoarthritis located in the medial compartment. Based on the ACR criteria, the severity of their degenerative changes was assessed by means of the Kellgren and Lawrence scale with the aid of current X-ray images taken within the past year—patients with stage II degeneration were included in the study. All of the participants provided their written consent to participate in the study. The exclusion criteria included clinically diagnosed conditions, injuries, or surgical interventions affecting balance disorders, specifically: the inability to walk independently without the use of orthopaedic aids such as crutches, a cane, or a walker; a history of surgical procedures and injuries particularly affecting the spine and lower limbs; the presence of endoprosthesis; congenital deformities of the lower limb joints; knee joint infections; rheumatic diseases such as rheumatoid arthritis, ankylosing spondylitis, and psoriatic arthritis; degenerative joint diseases other than in the knee joints; neurological deficits (e.g., epilepsy, Parkinson’s disease, polyneuropathy, acute sciatica); vestibular and inner ear disorders; significant uncorrected and untreated visual impairments; insomnia; advanced ischemic heart disease; peripheral artery disease; the use of medications that may impair psychophysical performance; persistent foot and ankle dysfunctions or deformities affecting knee joint alignment (e.g., severe flat feet or ankle varus deformity resistant to correction). After taking into account the inclusion and exclusion criteria, 125 people were included in the study.

The participants were divided into two groups: a control group (*n* = 125) consisting of healthy individuals and a study group (*n* = 125)—the patients diagnosed with gonarthrosis. The demographic characteristics of the group are presented in Table 1. 

During the qualification and functional assessment of the patients, a subjective examination was conducted, which included the patient’s data, BMI, and the type of changes observed in X-ray examinations. A detailed interview was conducted to determine whether the patient qualified for further examination. The level of pain was determined using the Visual Analog Scale (VAS). Subsequently, a physical examination was performed, including a manual assessment of the knee joint using functional tests to determine the condition of the articular surfaces in the patellofemoral and tibiofemoral joints, the degree of meniscal damage, and their ligamentous apparatus’ endurance, aiming to exclude other dysfunctions in the knee joint area. Next, the functional assessment was carried out for the patient by means of the following tests: the “Up and Go” Test, Functional Reach Test, Five Time Sit to Stand Test, and Step Test. The Up and Go test allows you to assess your balance when getting up from a chair, walking, and sitting down. From a sitting position on a 46 cm high chair, the patient gets up and covers a distance of 3 m, and then turns around and returns to their starting point, sitting on a chair. The test was performed bilaterally for the right and left sides. A transition time of less than 10 s is considered normal. While walking, the patient also assessed the pain in their knee joints on a VAS scale from 0—no pain to 10—unbearable pain. In the Functional Reach Test, the patient stands in the starting position with their arms raised in front of them, bent to 90 degrees at the shoulder joints, and performs a forward movement of the torso within the range of their body’s control. The time the tilt position is maintained for and the distance are measured, and the pain in the area of the knee joint is measured using the VAS scale. The patient cannot lift their heels off the ground or lose balance. In the Five Time Sit to Stand Test, a patient must stand up 5 times in the shortest possible time and sit on a 46 cm high chair with their arms crossed on their chest. This test is used to functionally assess lower limb muscle strength and balance. In turn, the Step Test measures the number of times one lower limb can climb a 7.5 cm step in 15 s.

Then, an assessment was conducted on a FreeMED Base tensometric platform, using the Free Step vs. 1.3.5 software, by means of the posturographic test—the Romberg test. This is performed with eyes open (OE) and closed (CE) in a standing position, with arms raised in front and feet spaced apart by the width of a fist for 50.1 s. The parameters determining the centre of gravity (COG), represented by the measured centre of foot pressure (COP), including the sway length, ellipse area, mean velocity, and maximum and minimum sway, were evaluated. The obtained parameters for the balance assessment were also evaluated clinically by means of the Berg Balance Scale. All functional tests were performed at least three times. The average score was given as the evaluation. The data obtained were analysed with the use of the IBM SPSS Statistics software version 27.0 by means of the Mann–Whitney test in order to compare the changes in measurements between the control group and the study group, and correlations were determined by means of Spearman’s test. A significance level of *p* = 0.05 was adopted.

### Outcome

Significant statistical differences were observed between the assessed groups on the VAS scale both at rest, during exertion, at night, and during the day (*p* < 0.001). In the control group, individuals did not report any knee joint pain. The obtained data suggest that, during exertion, joint pain was greater than at rest, and during the day it was greater than at night, which aligns with the typical symptoms reported by patients with knee osteoarthritis.

In the Up and Go Test, statistically significant differences were obtained for the assessment of the time taken and pain (*p* < 0.001). Patients in the study group took a longer time and experienced more pain during the test compared to the individuals in the control group. The patients with knee osteoarthritis demonstrated worse parameters compared to the healthy individuals, which was also evident in the Forward Lean Test—the healthy individuals achieved better results, and the observed differences were statistically significant. There were no differences observed in the distance of the forward lean between the groups, only in the assessment of pain and the time the forward leaning position was maintained for. From the presented tests, it is evident that the individuals in both the control and study groups have a similar range of motion and soft tissue flexibility, but they differ in their endurance—in terms of maintaining positions and experiencing pain—which should be addressed throughout the course of rehabilitation training. In the Five Time Sit to Stand Test, statistically significant differences were obtained (*p* < 0.001). The healthy individuals performed the task faster than those with degenerative changes in their knee joints. Similarly, differences were demonstrated between the groups in the number of steps taken with their right and left feet in the Step Test—higher scores were observed in the control group than in the study group. This may indicate a better physical fitness among the individuals in that group (Table 2).

In the posturographic examination, worse results were observed for the Romberg test in the study group compared to the control group, as far as the median was concerned, for the following parameters: sway length, ellipse area, mean velocity, and maximum and minimum sway values with eyes open and closed. However, this was not the case for the parameter evaluating the ellipse area with eyes closed (*p* = 0.786). For all the evaluated parameters, except for the ellipse area with eyes closed, significant differences were obtained between the groups. Those results clearly indicate that the patients with knee osteoarthritis exhibit deficits in their balance, as expressed in the posturographic examination conducted by means of the Romberg test (Table 3).

Similarly, statistically significant differences between the patients and the healthy individuals (*p* < 0.001) were obtained in the assessment of their balance using the Berg Balance Scale. This allows us to conclude that the individuals with degenerative changes face difficulty in maintaining balance as measured by the Berg Balance Scale (Table 4). Furthermore, comparing the posturographic examination to the functional Berg Balance Scale, significant differences were observed in the Romberg test between the control and study groups in the assessment of their minimum and maximum values, median, and mean. Similar observations can be made when comparing posturography to the functional assessment. This makes it plausible to state that the posturographic examination evaluates the risk of falls better than the Berg Balance Scale. 

The obtained results confirm this hypothesis, as individuals in the early stage of gonarthrosis were assessed (those not included in the study were those who had qualified for surgical procedures), and it was shown that both in the posturographic examination and in the functional tests, as well as in the assessment using the Berg Balance Scale, the study group significantly differed from the control group. This allows us to conclude that deficits in lower limb function are observed in patients with stage II gonarthrosis of the knee joints. Their lower limb dysfunction was expressed in both the clinical examination and posturographic examination, with the posturographic examination appearing to be a more precise assessment method than the regular functional assessment. The obtained results were also evaluated in terms of correlations with characteristics such as the duration of symptoms, age, and BMI (Table 5).

Statistically significant negative correlations were obtained for the duration of symptoms with variables such as the Berg Balance Scale, distance and time in the forward lean test, and the number of steps taken with the right and left lower limb in the Step Test. These correlations indicate that the longer the duration of the disease, the lower the values of the above parameters. On the other hand, positive correlations were obtained for the assessment of pain levels: pain at rest, during exertion, during the day, and at night; time to complete the passage of the right and left side in the Up and Go Test; pain level during its execution; pain during the Five Time Sit to Stand Test; and the level of pain during the forward lean. Additionally, positive correlations were obtained from the posturographic examination using the Romberg test for the length of sway, average speed with closed and open eyes, and maximum and minimum sway with open eyes. This confirms that the longer the patient has been ill, the worse their functionality.

In relation to the BMI assessment, there were few correlations suggesting that, as body weight increases, the level of balance measured by the Berg Balance Scale decreases. There is also a deterioration in the forward lean time with increasing body weight. Additionally, a positive correlation was observed when assessing the intensity of pain during the day and during the transition from sitting to standing—overweight and obese patients had greater difficulty in quickly completing the task of changing positions in a short period of time, and they experienced more pain as their weight increased. A higher body mass leads to greater stress on the knee joints, thus increasing pain symptoms, which affects functional efficiency. 

## 3. Discussion

Gonarthrosis is a pathological process involving the destabilisation of the degradation and synthesis processes of the articular cartilage, ultimately leading to disability [12,14]. However, the mechanism of these degenerative changes arises from the overlapping of mechanical and biological factors. Excessive overload, resulting from a reduced articular cartilage surface and unevenly distributed loads acting on the joint, is particularly noteworthy. This leads to increased pressure on the articular surfaces, causing a faster degeneration of the diseased cartilage, triggering pain and affecting the function of the lower limb’s biokinetic chain [15,16]. Therefore, individuals with osteoarthritis experience a loss of proprioception in addition to joint changes, which directly affects their ability to maintain posture and increases their risk of falls [17]. Numerous studies indicate that a decreased tolerance for loading and reduced physical activity due to the pain associated with aging lead to muscle volume reduction and joint instability. Therefore, it is plausible to state that chronic pain plays a significant role in the development of muscle atrophy and the imbalance between antagonistic muscle groups, disrupting biomechanics and significantly limiting a person’s range of motion. As a result of these processes, changes occur in the surrounding tissues, further triggering nociceptive stimuli [10,18,19]. The latest reports suggest that deep muscles, including the diaphragm, which is rich in pressure-sensitive receptors, play a role in balance. They are activated during respiratory work by the pressure generated in the abdominal cavity [32,33]. Moreover, those muscles aid in maintaining the spine in its most stable position, which contributes to a better control of body movements and posture [34,35]

Scientific reports suggest that risk factors such as balance disturbances and muscle weakness are responsible for incidents of falls in patients with knee osteoarthritis. Unfortunately, the evidence is limited, and inconsistencies in previous studies indicate the need for further assessment to determine which factors are significant. Manlapaza et al. indicate that the risk factors for falls in individuals with knee osteoarthritis may also include the presence of comorbidities and joint pain. However, the strength of this evidence is too weak and inconsistent findings are provided. Limited reports have been found regarding knee joint instability, impaired proprioception, and the use of walking aids. Identifying risk factors is valuable in fall prevention. Further research is needed to determine which risk factors for falls can be modified in the population with knee osteoarthritis [36]. On the other hand, the study conducted by Chaharmahali et al. suggests that instability is a common problem in patients with knee osteoarthritis, affecting their daily functioning and balance. A study has aimed to assess the state of postural control in women with knee osteoarthritis with and without knee instability. Their static and dynamic balance was evaluated with open and closed eyes by means of a Biodex balance device, and their foot pressure distribution was measured by means of an FDM-S-Zebris device. Based on the results obtained, significant differences were found between the mean scores for pain, static and dynamic balance, and the risk of falls among the women with knee osteoarthritis with and without instability. Therefore, patients with knee osteoarthritis with instability are more prone to falls and require the use of appropriate management strategies [37]. Indeed, the fact that balance disorders are observed in patients with degenerative changes in their knee joints is also confirmed by a study conducted by Truszczyńska-Baszak et al., which showed that pain associated with severe knee osteoarthritis could lead to static balance disorders. Balance measurements were carried out by means of a CQStab2P platform. In the study group, there was greater asymmetry in the distribution of balance between the lower limbs compared to the control group. The patients tended to unload painful and unstable joints. Statistically significant differences were observed with their eyes open and closed [38].

In this study, individuals with gonarthrosis underwent both clinical and posturographic examinations. The obtained results were compared with a control group consisting of healthy individuals. Statistically significant differences were obtained for all the evaluated clinical aspects such as pain level and the time taken and/or pain level in tests—the Up and Go, forward lean, sit-to-stand transitions—and in the number of steps taken while walking up stairs. Differences between the groups were also observed in the functional assessment, according to the Berg Balance Scale and posturographic test—Romberg test—for parameters such as the length of sways, ellipse area, centre of gravity displacement, speed, and minimal and maximal sways.

In the study conducted by Khalaj N. et al., it was demonstrated that balance was essential for mobility and performing daily activities, and individuals with gonarthrosis exhibited poorer joint proprioception compared to healthy individuals. Individuals with bilateral gonarthrosis to mild or moderate degrees were compared in terms of a balance assessment and for their fall risk by means of the Biodex Stability System and the Up and Go Test. Significant differences in balance (both dynamic and static) were found between the control group and the groups of patients with osteoarthritis, with the most significant difference observed between the healthy group and those with moderate degenerative changes in their knee joints. Significant differences in the clinical balance assessment in the case of the Up and Go Test were also observed among all three groups. Additionally, it was noted that foot positioning, as measured by the Biodex Stability System, influenced balance maintenance [17]. 

Similar observations were obtained in this study with respect to both dynamic balance, assessed with the Up and Go Test, and static balance, assessed posturographically on a force plate. The assessment also included patients with stage II osteoarthritis of the knee joint located in the medial compartment.

On the other hand, the primary objective of the study conducted by Taglietti M. et al. was to determine whether changes in the COP (Centre of Pressure) represented significant differences compared to other groups of the patients with knee osteoarthritis and healthy volunteers. Their secondary objective was to compare the COP values of older women with knee osteoarthritis with those of the control group, in a bipedal stance with eyes open and closed. For this purpose, their static postural balance was measured by means of a force plate. It was found that the women with knee osteoarthritis had greater postural sway compared to the control group when standing with their eyes open [39]. 

Similar observations were obtained in this study—the individuals with knee osteoarthritis showed greater ranges in the assessment of the length of their sways, ellipse area, or the speed of their centre of gravity displacement compared to healthy individuals.

## 4. Conclusions

This study’s outcome has revealed disturbances in the balance, functionality, and posture of patients with knee osteoarthritis compared to healthy individuals in a control group. These findings allow for the following conclusions to be drawn:

- Patients with stage II osteoarthritis of the knee achieve poorer results in both clinical–functional assessments and posturographic examinations compared to healthy individuals, suggesting a correlation between the presence of degenerative changes in the knee joints and disturbances in balance and proprioception;
- Posturographic examination appears to be a reliable, sensitive, and objective research method for evaluating balance disorders in individuals with knee osteoarthritis, allowing researchers to plan relevant therapeutic strategies.

**Limitation, recommendations, and generalizations:** The research carried out had limitations as well as benefits. The limitations include the fact that the results of the posturgraphic and functional tests could be influenced by the patient’s state of rest or mood, which could lower the test values. In a future study, the inclusion of an additional survey assessing the patient’s mood and wakefulness should be considered. Additionally, the patients’ level of physical activity may also have an impact on their ability to maintain balance, which should be taken into account in further research. The presented posturographic and clinical assessments can be used in the offices of both primary care physicians and physiotherapists to detect balance disorders in people with early gonarthrosis, which is one of the advantages of this test.

## Figures and Tables

**Table 1:** Demographic characteristics of study groups.

		Variable	n	M	Me	Min	Max	Q1	Q3	SD
Control Group	Whole Group	Age	125	51.88	55.00	28.00	63.00	48.00	59.00	9.32
		Duration of symptoms	125	5.33	3.50	0.25	25.00	2.00	7.00	5.06
		BMI	125	27.65	26.85	18.52	39.79	24.16	29.83	4.54
	Women	Age	94	53.60	55.00	30.00	63.00	52.00	60.00	7.80
		Duration of symptoms	94	5.02	3.00	0.25	25.00	1.50	7.00	5.06
		BMI	94	26.98	26.29	18.52	39.79	23.80	29.05	4.31
	Men	Age	31	46.68	44.00	28.00	62.00	33.00	57.00	11.53
		Duration of symptoms	31	6.26	4.50	0.50	20.00	3.50	7.50	5.05
		BMI	31	29.71	28.60	22.72	37.51	24.86	32.98	4.68
Study Group	Whole Group	Age	125	40.16	36.00	30.00	60.00	32.00	47.00	9.79
		Duration of symptoms	125	0.00	0.00	0.00	0.00	0.00	0.00	0.00
		BMI	125	27.14	25.98	18.94	38.67	23.84	30.04	5.30
	Women	Age	74	40.69	33.50	30.00	60.00	32.00	48.00	10.86
		Duration of symptoms	74	0.00	0.00	0.00	0.00	0.00	0.00	0.00
		BMI	74	26.75	25.10	19.38	38.67	20.82	33.06	5.80
	Men	Age	51	39.39	36.00	30.00	58.00	32.00	45.00	8.03
		Duration of symptoms	51	0.00	0.00	0.00	0.00	0.00	0.00	0.00
		BMI	51	27.71	27.47	18.94	34.87	25.68	29.76	4.48

**Table 2:** Comparison between the control group and the study group that underwent clinical tests.

	Control Group							Study Group							
	M	SD.	Min	Q1	Me	Q3	Max	M	SD	Min	Q1	Me	Q3	Max	*p*
VAS	0.00	0.00	0	0	0	0	0	5.08	1.73	1	4	5	6	8	0.000
Resting Pain	0.00	0.00	0	0	0	0	0	2.76	2.56	0	0	2	4	9	0.000
Pain During Exertion	0.00	0.00	0	0	0	0	0	5.17	2.29	0	4	5	7	10	0.000
Pain During the Day	0.00	0.00	0	0	0	0	0	4.40	2.13	0	3	5	6	8	0.000
Pain During the Night	0.00	0.00	0	0	0	0	0	2.47	2.78	0	0	2	5	9	0.000
Up and Go Test—Time (Right Side)	6.32	0.97	5.2	5.7	6.1	6.6	10	7.79	1.51	4	6.98	7.5	8.51	12	0.000
Up and Go Test—Time (Left Side)	6.24	0.94	5.2	5.7	6	6.5	9.1	7.93	1.52	5	6.89	7.7	9.12	12	0.000
Up and Go Test—Pain (Right Side)	0.00	0.00	0	0	0	0	0	1.13	1.58	0	0	0	2	5	0.000
Up and Go Test—Pain (Left Side)	0.00	0.00	0	0	0	0	0	0.98	1.54	0	0	0	2	5	0.000
Functional Reach Test—Length	33.58	5.45	19	30	33	37	47	31.54	7.37	11	27	32	36	45	0.162
Functional Reach Test—Time	31.68	12.07	8	24	30	37, 5	58	18.50	12.19	4	8	15	25	60	0.000
Functional Reach Test—Pain	0.02	0.16	0	0	0	0	1	1.58	2.22	0	0	0	3	8	0.000
Five Time Sit Test	8.35	3.99	5.005	6.0275	6.995	9.39	27.5	12.39	3.01	5.915	10.81	11.74	13.26	20.585	0.000
Step Test for Right Foot	18.93	2.57	10	18	19	20.5	22	13.40	2.79	9	11	13	15	22	0.000
Step Test for Left Foot	19.27	3.15	10	18	20	21	29	13.62	2.93	9	11	13	16	22	0.000

**Table 3:** Comparison between the control and study group in terms of the posturographic examination—the Romberg test.

			Control Group					Study Group							
	M	SD	Min	Q1	Me	Q3	Max	M	SD	Min	Q1	Me	Q3	Max	*p*
Sway Length OE	1078.89	218.95	758.35	907.465	1034.27	1205.525	1733.3	272 1.85	779.77	146 2.01	2085.0425	2548.16	3187.33	4960.47	0.000
Sway Length CE	1153.66	269.69	763.45	945.145	1118.76	1285.25	1692.02	260 6.27	715.19	133 4.03	2194.8	2514.475	2876.73	5693.17	0.000
Ellipse Area OE	149.46	208.13	14.2	50.645	80.24	119.46	909.75	175.35	188.85	23.56	69.0375	103.675	192.89	1098.68	0.030
Ellipse Area CE	118.17	91.77	11.59	53.99	90.73	172.755	414.07	140.00	132.81	3.55	49.3225	86.02	185.835	648.27	0.786
Mean Seed OE	21.23	4.32	14.99	17.775	20.31	23.71	34.14	53.64	15.27	28.73	41.51	50.09	62.61	97.48	0.000
Mean Speed CE	22.57	5.32	14.91	18.405	21.75	25.1	33.1	51.61	14.34	25.85	43.21	49.99	58.89	111.64	0.000
Max Sway OE	1.33	0.27	0.96	1.11	1.32	1.515	2.36	2.57	0.60	1.57	2.1875	2.445	2.87	4.05	0.000
Max Sway CE	5.38	4.78	1.28	2.08	3.69	5.015	18.7 9	6.72	5.01	1.75	3.035	5.24	8.75	26.67	0.032
Min Sway OE	0.00	0.01	0	0	0	0.01	0.02	0.01	0.01	0	0.01	0.01	0.01	0.02	0.000
Min Sway CE	0.01	0.01	0	0	0	0.01	0.02	0.01	0.01	0	0	0.01	0.01	0.02	0.012

**Table 4:** Comparison between the control group and the study group in terms of the Berg Balance Scale.

			Control Group							Study Group					
	M	SD	Min	Q1	Me	Q3	Max	M	SD	Min	Q1	Me	Q3	Max	*p*
Berg Balance Scale	55.88	0.56	53	56	56	56	56	54.84	1.58	49	54	55	56	56	0.000

**Table 5:** Correlation values for age, duration of symptoms, and BMI.

	Duration of Symptoms		Age		BMI	
	r	*p*	r	*p*	r	*p*
Berg Scale	−0.44	0.00	−0.39	0.00	−0.49	0.00
VAS	0.42	0.00	0.23	0.00	0.08	0.33
Resting Pain	0.20	0.01	0.27	0.00	−0.18	0.02
Pain During Exertion	0.46	0.00	0.43	0.00	0.19	0.01
Pain During the Day	0.49	0.00	0.30	0.00	0.17	0.03
Pain During the Night	0.36	0.00	0.37	0.00	0.00	0.97
Up and Go Test—Time (Right Side)	0.45	0.00	0.41	0.00	0.05	0.52
Up and Go Test—Pain (Right Side)	0.28	0.00	0.49	0.00	0.03	0.67
Up and Go Test—Time (Left Side)	0.48	0.00	0.47	0.00	0.05	0.50
Up and Go Test—Pain (Left Side)	0.36	0.00	0.39	0.00	−0.07	0.36
Functional Reach Test—Length	−0.21	0.01	−0.41	0.00	0.00	0.99
Functional Reach Test—Time	−0.38	0.00	−0.53	0.00	−0.24	0.00
Functional Reach Test—Pain	0.31	0.00	0.18	0.02	0.25	0.00
Five Time Sit Test	0.40	0.00	0.43	0.00	0.19	0.01
Step Test for Right Foot	−0.52	0.00	−0.46	0.00	−0.08	0.29
Step Test for Left Foot	−0.49	0.00	−0.45	0.00	−0.12	0.14
Sway Length OE	0.40	0.00	0.31	0.00	0.01	0.88
Sway Length CE	0.47	0.00	0.34	0.00	0.04	0.62
Ellipse Area OE	0.09	0.25	0.10	0.22	−0.10	0.20
Ellipse Area CE	0.08	0.32	0.15	0.05	−0.03	0.74
Mean Seed OE	0.40	0.00	0.31	0.00	0.01	0.86
Mean Seed CE	0.44	0.00	0.35	0.00	0.05	0.52
Max Sway OE	0.38	0.00	0.26	0.00	−0.03	0.69
Max Sway CE	0.08	0.32	0.03	0.73	−0.12	0.13
Min Sway OE	0.19	0.01	0.07	0.39	−0.08	0.29
Min Sway CE	0.08	0.33	0.00	0.96	0.07	0.39

## Data Availability

The data presented in this study are available on request from the corresponding author due to (The data presented in this study are made available at the request of the corresponding author due to the limitations of RODO data availability).
